# Bemoan My Collarbone: A Case of Costocondral Junction Syndrome Complicated by Methicillin Sensitive Staphylococcus aureus Sternoclavicular Osteomyelitis and Septic Arthritis

**DOI:** 10.7759/cureus.34108

**Published:** 2023-01-23

**Authors:** Vashistha M Patel, Shreya V Patel, Lauren Pacheco

**Affiliations:** 1 Internal Medicine, Brookwood Baptist Medical Center, Birmingham, USA

**Keywords:** teitze syndrome, costochondral junction syndrome, intravenous drug use (ivdu), clavicular osteomyelitis, mssa sternoclavicular septic arthritis

## Abstract

A 35-year-old female with a past medical history of untreated Hepatitis-C, and a history of intravenous (IV) drug use initially presented to the emergency department with chief complaints of gradual worsening sharp, constant left-sided chest pain with no radiation starting three weeks before presentation. In the emergency department (ED), she was afebrile, normotensive, and tachycardia with 99% oxygen saturation on room air. A physical exam revealed a well-developed Caucasian female, alert and oriented with moderate distress. Respiratory exam with symmetrical bilateral excursions without wheezes, crackles, or rhonchi. On cardiovascular exam, she was tachycardic with a regular rhythm without murmurs, rubs, or gallops. There was a 2 x 2 cm tender erythematous swelling on the left sternal border inferior to the clavicle. The neck was supple and negative for Jugular Venous Distension (JVD). Neurologically grossly intact. Abnormal laboratory findings included leukocytosis with neutrophilic predominance. The patient received intravenous (IV) antibiotics with broad-spectrum vancomycin, cefepime, and azithromycin and underwent computed tomography angiography (CTA) chest, revealing a 26.8 mm x 26.5 mm left anterior subapical pleural-based pulmonary mass-like lesion with central hypoattenuation in surrounding ground-glass changes. Biopsy of the left subapical pulmonary lesion results showed chronic inflammatory infiltrate. Unfortunately, the patient left the hospital against medical advice after supportive care and pain control. Our patient's history of intravenous drug use and active Hepatitis-C infection were typical risk factors associated with invasive infections. In the clinical context, leukocytosis with hypo-attenuated pulmonary lesion should raise suspicion for septic emboli, localized abscess pocket, infection by atypical organisms, infective endocarditis, and malignancy which was considered upon initial assessment.

## Introduction

Medical literature shows that costochondral junction syndrome (Tietze syndrome) is a rare self-limiting condition that presents as pain and tenderness involving the sternocostal, sternoclavicular, or costochondral joints. Case reports indicate the treatment to be symptom control and steroid injections at the site of the lesion [1]. Costochondral syndrome disorder is an arthropathy treated conservatively with pain medications, applying ice or heat, and steroid injections [1]. We present an interesting case of Tietze syndrome complicated by Methicillin-sensitive *Staphylococcus aureus* infection of the costochondral junction and osteomyelitis of the left clavicular head.

## Case presentation

**Figure 1:**
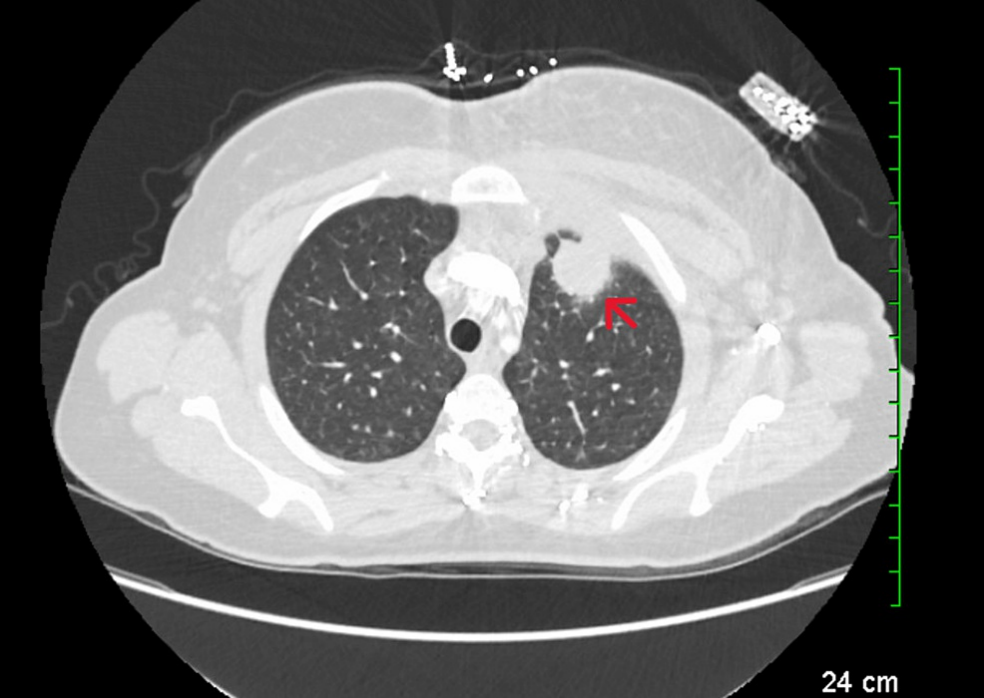
Initial CTA chest CTA: Computed tomography angiography The red arrow shows the sub-apical, mass-like lesion (26.8 mm x 26.5 mm)

A 35-year-old Caucasian female with a past medical history of untreated Hepatitis-C infection presented to the emergency department with three weeks of left-sided chest pain and chills. Her symptoms gradually progressed over three weeks. Upon further questioning, she stated worsening pain prompted her to visit the emergency department. She reported no recent sick contacts or recent hospitalizations or urgent care visits. She also denied any other associated symptoms such as dyspnea, palpitations, diaphoresis, cough, hemoptysis, hematemesis, abdominal pain, syncope, and changes in bowel and bladder habits. She had not received routine medical care before this visit. She was also not taking any home medications. She denied alcohol and tobacco use but endorsed intravenous (IV) drug use (methamphetamine) for the past few months. She was born in the United States and has never traveled outside the country. On presentation, she was normotensive at 128/66 mmHg with tachycardia at 107, afebrile at 98°C, respiratory rate at 18, and oxygen saturation at 99% on room air. She was alert and oriented but in moderate distress. A cardiovascular examination revealed tachycardia without any murmurs, rubs, or gallops. There was tender erythematous swelling on the left sternal border inferior clavicle. Respiratory system examination revealed symmetrical bilateral air movement without wheezes, crackles, or rubs. Neurological examination was unremarkable. There were no skin lesions observed. Abnormal laboratory findings included elevated white blood cell count at 12,500/mcl with a neutrophilic predominance (80.6%), anemia with hemoglobin 9.9 grams per deciliter (g/dl), and hematocrit 31.4. A basic metabolic panel revealed electrolytes within normal limits with serum creatinine at a baseline of 0.57 mg/dl. Chest X-ray revealed a vague rounded mass overlying the left lung apex. CTA chest revealed a 26.8 mm x 26.5 mm left anterior subapical pleural-based pulmonary mass-like lesion with central hypoattenuation and surrounding ground-glass changes (Figure 1).

Blood cultures were drawn followed by initiation of intravenous vancomycin, cefepime, and azithromycin. The patient's tachycardia improved with gentle IV fluids. She was administered nonsteroidal anti-inflammatory agents for pain control. Interventional Radiology was consulted for an ultrasound-guided biopsy which was performed on day two of admission. Unfortunately, on day three of admission the patient left the hospital against medical advice after the lesion subsided with supportive care and pain control. Eventually, pathology reports from biopsy samples showed chronic inflammatory infiltrate. Microbiology culture data from the biopsy sample showed no growth as well.

**Figure 2:**
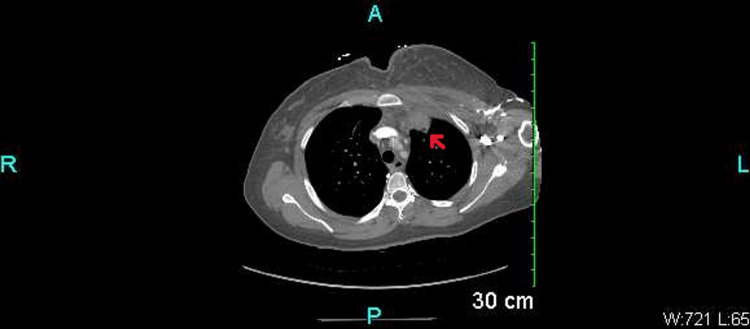
CT chest 3 weeks later The image shows a 33 mm x 33 mm new periosteal reaction and destructive bone loss.

Three weeks later the patient presented to the same hospital with recurrent symptoms of chest pain at the same location. She reported a flare-up of similar tender swelling. Again, on presentation, she was normotensive with a blood pressure of 110/63 mmHg with tachycardia at 110 and respiratory rate of 20, afebrile with a temperature of 99° C with oxygen saturation at 99% on room air. A physical exam revealed her to be in moderate distress with tachycardia otherwise cardio-pulmonary exam was normal. There was a recrudescence of tender erythematous swelling on the left sternal border inferior to the clavicle. Chest X-ray was consistent with a rounded mass overlying the left lung apex. CT chest revealed an increase in the size of the old lesion to 33 mm x 33 mm with a worsening inflammatory process involving the left Costochondral junction and medial clavicle. A new periosteal reaction along the left medial clavicular destructive bone loss of the anterior first rib was also found (Figure 2) [2,3].

Again, blood cultures were drawn before initiation of IV broad-spectrum vancomycin, cefepime, and azithromycin. After admitting her to the floor, Pulmonology and Cardiothoracic surgery were consulted. They planned for left thoracotomy with resection of the lesion. The patient underwent surgery the next day and had no major post-operative complications. Blood cultures were negative for any growth. Postoperative tissue culture came back positive for Methicillin Sensitive *Staphylococcus aureus *(MSSA). Imaging, intraoperative findings, and culture data supported a final diagnosis of septic arthritis of the sternoclavicular joint with associated clavicular osteomyelitis by MSSA. Infectious diseases were consulted and while inpatient she was administered IV nafcillin with a plan for weekly IV dalbavancin infusions for six weeks in total after discharge.

## Discussion

Clinical suspicion for septic emboli, localized abscess pocket, infection by atypical organisms, and malignancy were considered upon initial assessment. Biopsy, tissue culture, and imaging were the mainstay in definitive diagnosis. Septic arthritis of the sternoclavicular joint is rare and represents 1% of all joint infections. Some cartilaginous tissue receives blood supply via the mechanism of diffusion, while vascular supply to sternoclavicular tissue is derived from the internal thoracic artery and suprascapular artery. Due to the joint's anatomically deep position, the hematologic spread is rare without any evidence of an external injection site. A nearby bone infection can result in bacterial seeding resulting in the spread of infection. Concurrent medical conditions and patients with active infection, immunosuppression, intravenous drug use, and prior similar episodes should be kept under review for prompt diagnosis and treatment to prevent the spread of infection to the regional vasculature, mediastinum, lungs, and surrounding soft tissue. Mainstay treatment includes intravenous antibiotics and prompt surgical irrigation and debridement of the involved joint if required [4,5]. Patients at risk for this condition usually have other predisposing factors that suggest active infection, and immunosuppression. Thus, without proper diagnosis and treatment, the regional spread can cause significant morbidity. In this case, the MSSA infection extended from the sternoclavicular joint space into the nearby clavicle. Refractory cases may require resection of the medial clavicle with possible soft tissue coverage, given the subcutaneous nature of the sternoclavicular joint [5]. There is no strong evidence for reimaging patients on follow-ups. C-reactive proteins (CRPs) generally indicate an inflammatory state. Regular interval CRP checks are not recommended.

## Conclusions

Care should be taken to evaluate the surrounding chest wall for loculated abscesses that may be a source of continued infection at the time of admission in high-risk patients. When dissecting the medial clavicle, the vital retrosternal structures, as well as the surrounding ligamentous structures, must be protected. Damage to supporting ligamentous structures can result in chronic pain and joint instability. High suspicion for soft tissue infection at the atypical sites should be kept in patients with high-risk behaviors such as intravenous drug use, untreated infectious diseases, history of incarceration, and sexually transmitted infections (STI). We do need to treat patients with empiric antibiotics due to close proximity to the cardiopulmonary as well as the mediastinal vasculature. Antibiotics can be tailored once culture data is obtained. Prompt initiation of intravenous antibiotics with a low threshold for surgical consultation is a vital step in improving the overall outcome for the patients.
